# The *LDLR* c.501C>A is a disease-causing variant in familial hypercholesterolemia

**DOI:** 10.1186/s12944-021-01536-3

**Published:** 2021-09-12

**Authors:** Haochang Hu, Ruoyu Chen, Yingchu Hu, Jian Wang, Shaoyi Lin, Xiaomin Chen

**Affiliations:** 1 School of Medicine, Ningbo University, Ningbo, Zhejiang, China; 2 Department of Cardiology, Ningbo First Hospital, Ningbo, Zhejiang, China

**Keywords:** Familial hypercholesterolemia, Genetic diagnosis, Whole-exome sequencing, Heterogeneous genotype, Homogeneous genotype, PCSK9 inhibitor

## Abstract

**Background:**

As an autosomal dominant disorder, familial hypercholesterolemia (FH) is mainly attributed to disease-causing variants in the low-density lipoprotein receptor (*LDLR*) gene. The aim of this study was to explore the molecular mechanism of *LDLR* c.501C>A variant in FH and assess the efficacy of proprotein convertase subtilisin kexin type 9 (PCSK9) inhibitor treatment for FH patients.

**Methods:**

The whole-exome sequencing was performed on two families to identify disease-causing variants, which were verified by Sanger sequencing. The function of *LDLR* variant was further explored in HEK293 cells by Western Blot and confocal microscopy. Besides, the therapeutic effects of PCSK9 inhibitor treatment for two probands were assessed for 3 months.

**Results:**

All members of the two families with the *LDLR* c.501C>A variant showed high levels of LDLC. The relationship between the clinical phenotype and *LDLR* variants was confirmed in the current study. Both *in silico* and *in vitro* analyses showed that *LDLR* c.501C>A variant decreased LDLR expression and LDL uptake. PCSK9 inhibitor treatment lowered the lipid level in proband 1 by 24.91%. However, the treatment was ineffective for proband 2. A follow-up study revealed that the PCSK9 inhibitor treatment had low ability of lipid-lowering effect in the patients.

**Conclusions:**

*LDLR* c.501C>A variant might be pathogenic for FH. The PCSK9 inhibitor therapy is not a highly effective option for treatment of FH patients with *LDLR* c.501C>A variant.

**Supplementary Information:**

The online version contains supplementary material available at 10.1186/s12944-021-01536-3.

## Introduction

Familial hypercholesterolemia (FH, MIM#143890) is a common autosomal hereditary disease. The main clinical manifestations of FH are increased low-density lipoprotein cholesterol (LDLC) in the blood, xanthoma, corneal bow, and atherosclerotic cardiovascular disease (ASCVD) [1, 2]. For heterozygous FH (HeFH) and homozygous FH (HoFH) patients, the plasma LDLC levels are ≥ 190 mg/dL and ≥ 400 mg/dL, respectively [3]. Due to the long-term exposure to high LDLC from birth, FH patients might suffer from an increased risk of ASCVD and even myocardial infarction [4]. Recent studies have shown that the incidence of HoFH is about 1/300,000, and the incidence of HeFH is about 1/200-1/250 [5, 6]. Such a high incidence is bound to cause great health concerns and increase the medical care burden on society.

FH is caused by disease-causing variants in genes related to LDLC metabolism. Current research is mainly focused on four of the genes: low-density lipoprotein receptor (*LDLR*), low-density lipoprotein receptor adapter protein 1 (*LDLRAP1*), proprotein convertase subtilisin kexin 9 (*PCSK9*), and apolipoprotein B (*ApoB*)] [7]. The *LDLR* gene is the most frequently mutated gene in FH patients. There are at least 3,700 variants located in *LDLR* gene. These variants may directly lead to functional impairment or deletion of corresponding functional regions of LDLR [8]. A study of the Greek population identified 41 variants in the *LDLR* gene, out of which *LDLR* c.81C>G, c.517T>C, c.858C>A, c.1285G>A, c.1646G>A, and c.1775G>A changes accounted for more than 80% of all the variants [9]. Some scholars conducted a systematic review of *LDLR* variants in Chinese population. They found that most of the variants on the *LDLR* gene in the Chinese population were located in exon 4, and nearly 60% were missense mutations [10]. However, only a few *LDLR* variants have been confirmed to be pathogenic. The function of many variants of the *LDLR* gene has not been investigated. Therefore, expanding the spectrum of *LDLR* variants and exploring their functions are of great significance in understanding the pathogenesis of FH.

The primary goal of FH treatment is to reduce the levels of LDLC and the risk of ASCVD. At present, a large number of lipid-lowering drugs have been used in the clinical treatment of FH, including statins, ezetimibe and PCSK9 inhibitors [11]. Monoclonal antibodies for inhibiting PCSK9 have become highly effective drugs for the treatment of elevated LDLC levels and the prevention of ASCVD in FH patients [12]. For instance, treatment of HeFH and HoFH patients with PCSK9 inhibitors could result in a decrease of LDLC by 56% and 38%, respectively [13]. However, various lipid-lowering drugs could not sufficiently reduce LDLC levels in some FH patients. Therefore, development of new lipid-lowering drugs and assessment of their effect on FH patients are very important.

In this study, two FH families were selected to explore disease-causing variants by whole-exome sequencing. The functions of the variants were also verified by *in silico* and *in vitro* studies. In addition, the lipid-lowering efficacy of PCSK9 inhibitors was evaluated in two probands.

## Material and methods

### Study population, sample collection, and clinical information

In this study, members of two families were enrolled in the Ningbo First Hospital from January 2020 to June 2020. Dutch Lipid Clinic Network (DLCN) diagnostic criteria was used to evaluate the participants [14, 15]. The families had 5 patients (1-I-2, 1-II-1, 2-II-1, 2-II-3, 2-III-1) with definite FH phenotype (DLCN score > 8 points). HoFH was diagnosed using any of the following criteria: (1) Two allelic mutations in the *LDLR, APOB, PCSK9* or *LDLRAP1* gene; (2) untreated LDLC > 13 mmol/L (500 mg/dL) or treated LDLC ≥ 8 mmol/L (300 mg/dL) and meet any of the following conditions: a. xanthoma of the skin or tendon before the age of 10; b. untreated LDLC level of both parents increased in line with HeFH [16].

Blood samples (5 ml) were collected from all the participants and stored in a refrigerator at -80°C. Clinical data (including age, gender, lipid profile, treatment, medical history, etc.) was obtained from their clinical records in the hospital or through direct inquiry at the time of sampling.

### Whole-exome sequencing

Genomic DNA was extracted from whole blood samples using Omega Blood DNA Kit (Omega bio-tek, Georgia State, USA). NanoDrop 2000 instrument (Thermo Fisher Scientific, Shanghai, China) was used to determine the concentration and purity of extracted DNA. High-throughput whole-exome sequencing for DNA samples from FH patients was accomplished with BGISEQ-500 platform. The Burrows-Wheeler aligner was used to align the generated sequences with the human reference genome hg19 [17]. High-confidence variants in the sequencing data were identified using bioinformatics tools.

### *In silico* analysis

The impact of the variants on the expression and activity of LDLR protein was predicted by two bioinformatic tools (MutationTaster and Polyphen-2).

### Sanger sequencing

To validate variants on *LDLR* gene, PCR products were sequenced through ABI Prism 3730 sequencer (Applied Biosystems, California, USA). The sequencing results were analyzed with Chromas software. The details of PCR were as described previously [18]. The primers used for amplification of the *LDLR* gene are as follows: *LDLR* (upstream) 5’-GTGGTCTCGGCCCATCCATCCCTGC-3’; *LDLR* (downstream) 5’-TGCGGCCACTCATCCGAGCCATCTT-3’ [19]. The final reaction mixture included 1 μL template DNA (200 ng), 8 μl ddH2O, 10 μl 2x PCR Master Mix, 0.5 μL forward/ reverse primer (10 μm).

### Cell culture and plasmid transfection

The HEK293 cells were cultured with DMEM medium in a 37°C incubator containing 5% CO_2._ The cell density was adjusted to 5×10^6^ cells/15ml 24 hours before transfection. The cells were divided into three groups for transfection (Mutation group: *LDLR* mutant plasmid was transfected into HEK293 cells; Wild-type (WT) group: *LDLR* wild-type plasmid was transfected into HEK293 cells; NC group: HEK293 cells were transfected with empty plasmid). Lipofectamine 2000 (Thermo Fisher Scientific, Shanghai, China) was used for transfection of the HEK293 cells. Six hours post-transfection, the cells were refreshed with DMEM medium containing 10% fetal bovine serum (FBS). After 48 hours of HEK293 cells transfection, *LDLR* expression was analyzed.

### Expression of *LDLR* variants

Total protein was extracted from HEK293 cells with the total protein extraction kit (Thermo Fisher Scientific, Shanghai, China). Total protein was quantified using the BCA quantification kit (Beyotime Biotechnology, Shanghai, China). The expression levels of LDLR were detected by Western Blot according to the standard procedure [20].

### Uptake of Dil-LDL

HEK293 cells carrying WT or mutated *LDLR* gene were incubated with 10μg/ml Dil-LDL for 4 hours. The cells were washed three times and then fixed with 4% paraformaldehyde. Leica SPE confocal microscopy and ImageJ software were used to analyze the fluorescence signal in all the samples.

### The assessment of pathogenicity of the variants

American College of Medical Genetics and Genomics (ACMG) recommend the use of five standard terms to describe variants. These include " benign, likely benign, pathogenic, likely pathogenic, and uncertain significance" [21]. For pathogenic or likely pathogenic variants, the ACMG criteria divides each variant into supporting (PP1-5), moderate (PM1-6), strong (PS1-4) or very strong (PVS1) based on weight. These standards and guidelines were used in the interpretation of variants identified in this study.

### Statistical analysis

SPSS 24.0 software (SPSS Inc, Chicago, IL, USA) was used for analysis of all statistical data. The experimental results were plotted with GraphPad Prism 5 (GraphPad Software, La Jolla, CA). *P* < 0.05 was defined as statistically significant.

## Results

### The clinical information for two probands

#### Proband 1

**Figure 1:**
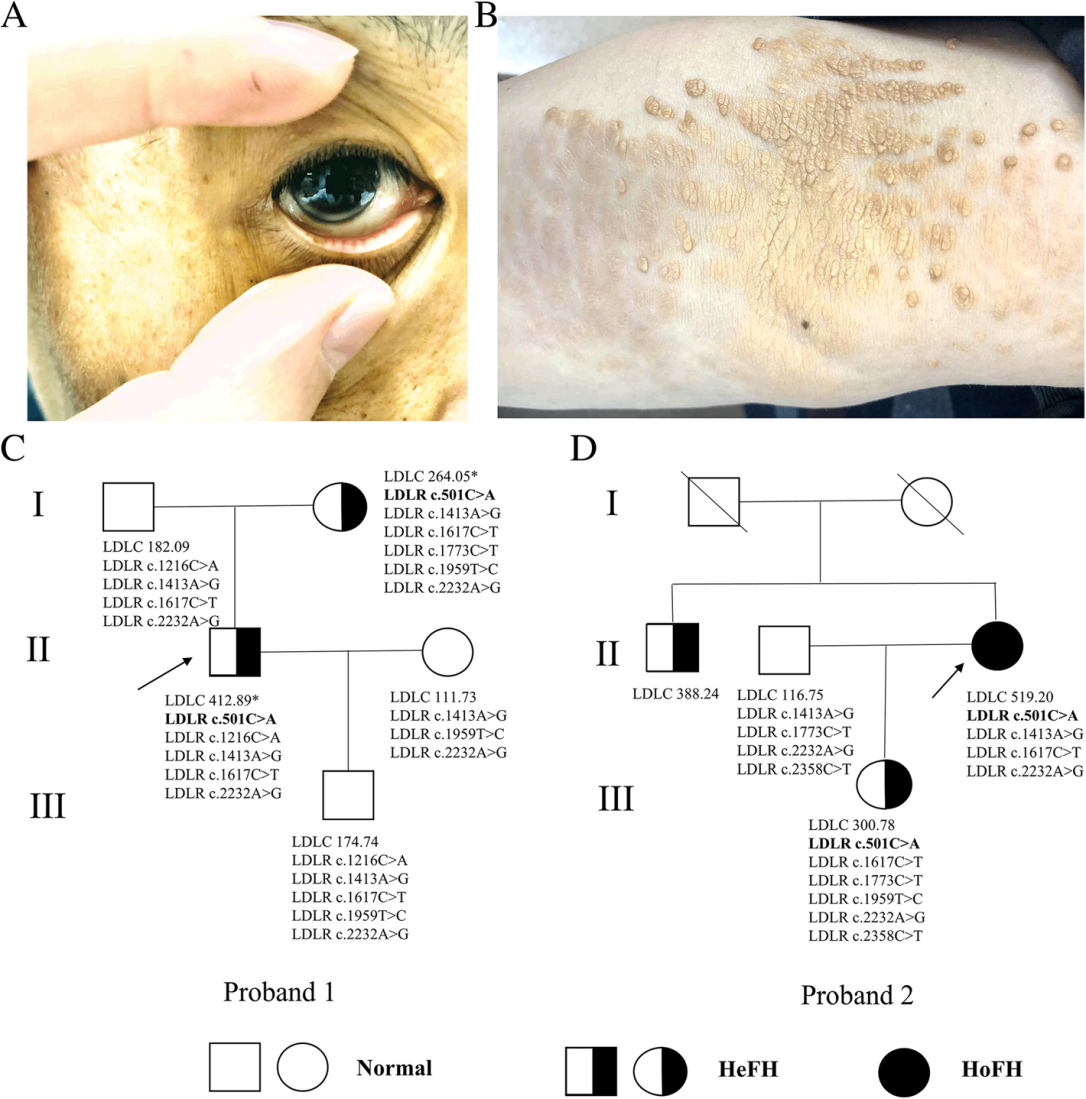
The corneal arch (A) and xanthoma (B) of proband 1. The family trees of two probands (C, D). * indicates that the patient is taking lipid-lowering drugs; → represents the proband. The disease-causing variants were bolded

A 41-year-old male from Ningbo suffered from premature coronary artery disease. Physical examination of the patient revealed corneal arch, xanthoma in elbow skin, neck skin, and tendon (Fig. 1A, 1B). The LDLC of proband 1 was 412.89 mg/dl (10.68 mmol/L), total cholesterol was 558.64 mg/dl (14.45 mmol/L), high-density lipoprotein cholesterol (HDLC) was 79.25 mg/dl (2.05 mmol/L), triglyceride was 101.89 mg/dl (1.15 mmol/L) under the treatment of 20 mg rosuvastatin and 10 mg ezetimibe per day (Data on blood lipids before medication was lost). The results of coronary angiography showed long diffuse stenosis in the left anterior descending coronary artery with a degree of 70%. Additionally, there was localized stenosis in the middle segment of the diagonal branch with a degree of 80%. Multiple plaques and a degree of 60% localized stenosis were also detected in the right coronary artery. The results of echocardiography suggested calcification of focal aortic valve with mild stenosis and regurgitation. The results also revealed localized calcification of the posterior mitral valve annulus and enlarged left atrium. Carotid ultrasound revealed bilateral bifurcation stenosis of common carotid artery (< 50%). The analysis also detected plaque formation in the right subclavian artery. Additionally, the bilateral carotid artery of the patient was thickened due to plaque build-up.

According to the DLCN diagnosis criteria for FH, a clinical diagnosis of definite FH (DLCN > 8 points) was made for proband 1.

#### Proband 2

A 48-year-old woman underwent coronary artery bypass surgery in Ningbo First Hospital. The patient had clinical manifestations of corneal arch, xanthoma in elbow skin, and neck skin. The LDLC of proband 2 was 548.59 mg/dl (14.19 mmol/L), total cholesterol was 687.38 mg/dl (17.78 mmol/L), triglyceride was 273.77 mg/dl (3.09 mmol/L), HDLC was 89.31 mg/dl (2.31 mmol/L) before medication. The results of echocardiography suggested calcification of the aortic valve with severe stenosis and mild regurgitation. The analysis also revealed large plaques on the wall of the aortic root and moderate mitral regurgitation. Additionally, the patient had an enlarged left atrium and thickened left ventricular wall. Carotid ultrasound revealed severe stenosis at the bifurcations of the bilateral internal carotid arteries and the right common carotid artery (> 70%). The analysis also revealed that the bilateral carotid artery intima-media of the patient was thickened with multiple plaques.

A clinical diagnosis of HoFH was made for Proband 2.

### Pedigree investigation

A total of 4 family members of proband 1 and 5 family members of proband 2 were included in our study. The pedigree maps of the two families are shown in Fig. 1C, 1D. The clinical and biochemical data of all participants are shown in Table 1. In the first family, the mother of proband 1 also had a relatively high LDLC. Both the proband 1 and his mother had a history of corneal arch, carotid plaque, and coronary heart disease. Therefore, proband 1 and his mother (1-I-2, 1-II-1) could be clinically diagnosed as FH (DLCN > 8 points). Notably, the 15-year-old son of proband 1 (1-III-1) had a higher lipid level than peers, and may also be a FH patient.

**Table 1:** Clinical data of FH patients and family members

Characteristics	1-I-1	1-I-2	1-II-1	1-II-2	1-III-1	2-II-1	2-II-2	2-II-3	2-III-1
Gender	Male	Female	Male	Female	Male	Male	Male	Female	Female
Age (year)	55	54	41	39	15	51	52	48	27
Triglycerides (mg/dL)	57.59	124.04	101.89	42.53	49.62	170.11	75.31	273.77	96.57
Total cholesterol (mg/dL)	255.54	378.10	558.64	186.34	271.39	477.06	159.28	687.38	379.64
High-density lipoprotein cholesterol (mg/dL)	55.28	76.93	79.25	61.08	74.23	71.91	41.37	89.31	58.76
Low-density lipoprotein cholesterol (mg/dL)	182.09	264.05	412.89	111.73	174.74	388.24	116.75	548.59	300.78
ApoA1 (g/L)	1.41	1.67	0.70	1.46	1.54	/	/	0.87	/
ApoB (g/L)	1.32	1.82	2.81	0.83	1.18	/	/	2.57	/
Lipoprotein a (mg/dL)	10.00	20.92	15.46	10.71	10.42	/	/	12.10	/
Carotid plaque	Yes	Yes	Yes	No	No	/	Yes	Yes	Yes
Carotid stenosis	No	No	Yes	No	No	/	No	Yes	No
Aortic valve Calcification	No	No	Yes	No	No	/	No	Yes	No
Left ventricular ejection fraction (%)	75	72	67	64	71	/	68	59	64
Corneal arch	No	Yes	Yes	No	No	Yes	No	Yes	No
Xanthoma	No	No	Yes	No	No	Yes	No	Yes	No

Through investigation of the family history, the parents of proband 2 were consanguineous. Both parents had high blood lipid levels. Unfortunately, their clinical data could not be obtained because they were deceased. The LDLC levels of the daughter and brother of proband 2 were also very high. By calculating DLCN scores, it was inferred that they (2-II-1, 2-II-3, and 2-III-1) all might suffer from FH (DLCN > 8 points). The above analysis revealed that the hypercholesterolemia in the two families might be hereditary.

### Whole-exome sequencing enables molecular diagnosis of FH

Molecular diagnosis of FH disease-causing variants for all the participants was performed using whole-exome sequencing. Seven FH-related genes (*LDLR, LDLRAP1, APOB, PCSK9, ABCG5/8* or *APOE*) were targeted for sequencing in this study [22]. Five variants of *LDLR* gene occurred in proband 1 namely c.501C>A, c.1216C>A, c.1413A>G, c.1617C>T and c.2232A>G. Four variants of *LDLR* gene were found in in the father of the proband 1, namely c.1216C>A, c.1413A>G, c.1617C>T and c.2232A>G. There were 6 variants of *LDLR* gene detected in the mother of the proband 1, namely c.501C>A, c.1413A>G, c.1617C>T, c.1773C>T, c.1959T>C and c.2232A>G. Three variants of *LDLR* gene were found in the wife of the proband 1, namely c.1413A>G, c.1959T>C and c.2232A>G. Five variants of *LDLR* were detected in the son of the proband 1, namely c.1216C>A, c.1413A>G, c.1617C>T, c.1959T>C and c.2232A>G (Fig. 1C).

For proband 2, four variants of *LDLR* gene were found, namely c.501C>A, c.1413A>G, c.1617C>T, and c.2232A>G. Four *LDLR* gene variants c.1413A>G, c.1773C>T, c.2232A>G and c.2358C>T were found in the husband of the proband 2. *LDLR* gene variants c.501C>A, c.1617C>T, c.1773C>T, c.1959T>C, c.2232A>G, and c.2358C>T were found in the daughter of proband 2 (Fig. 1D).

To confirm the function of the variants, *in silico* analysis of all the variants was performed. The results showed that *LDLR* c.1216C>A, c.1413A>G, c.1617C>T, c.1773C>T, c.1959T>C, c.2232A>G, and c.2358C>T variants were synonymous mutations. Interestingly, *LDLR* c.501C>A was a nonsense mutation, in which the 167 codon (TGC) for the amino acid cysteine (Cys) was replaced by the stop codon TGA (p.Cys167X). Therefore, the *LDLR* c.501C>A variation was identified as a pathogenic variant for FH. Moreover, the disease-causing variant *LDLR* c.501C>A was confirmed in the FH patients by Sanger sequencing (Supplement Figure 1).

It is worth noting that no pathogenic variant in the exons of *APOB, PCSK9, LDLRAP1, ABCG5/8* and *APOE* genes was found in these two families. And the data of proband 1 on all variants in *LDLR, APOB, PCSK9* genes with minor allele frequency (MAF) less than 0.05 were shown in the Supplementary Table 1.

### FH genotype was correlated with phenotype

According to the sequencing results, both proband 1 and his mother were heterozygous for the *LDLR* c.501C>A. The proband 2 was HoFH, but his daughter was HeFH. However, the LDLC of the brother of proband 2 was high (388.24 mg/dL). According to the DLCN score, he should also be diagnosed as FH. Unfortunately, his sample cannot be obtained for DNA. The incidence of ASCVD was higher in FH patients with the *LDLR* c.501C>A variant than that of non-FH patients in the family. In addition, the HoFH (2-II-3) patient had more severe clinical manifestations compared with the 4 HeFH patients.

### *LDLR* c.501C>A variant prevents the expression of the LDLR protein

**Figure 2:**
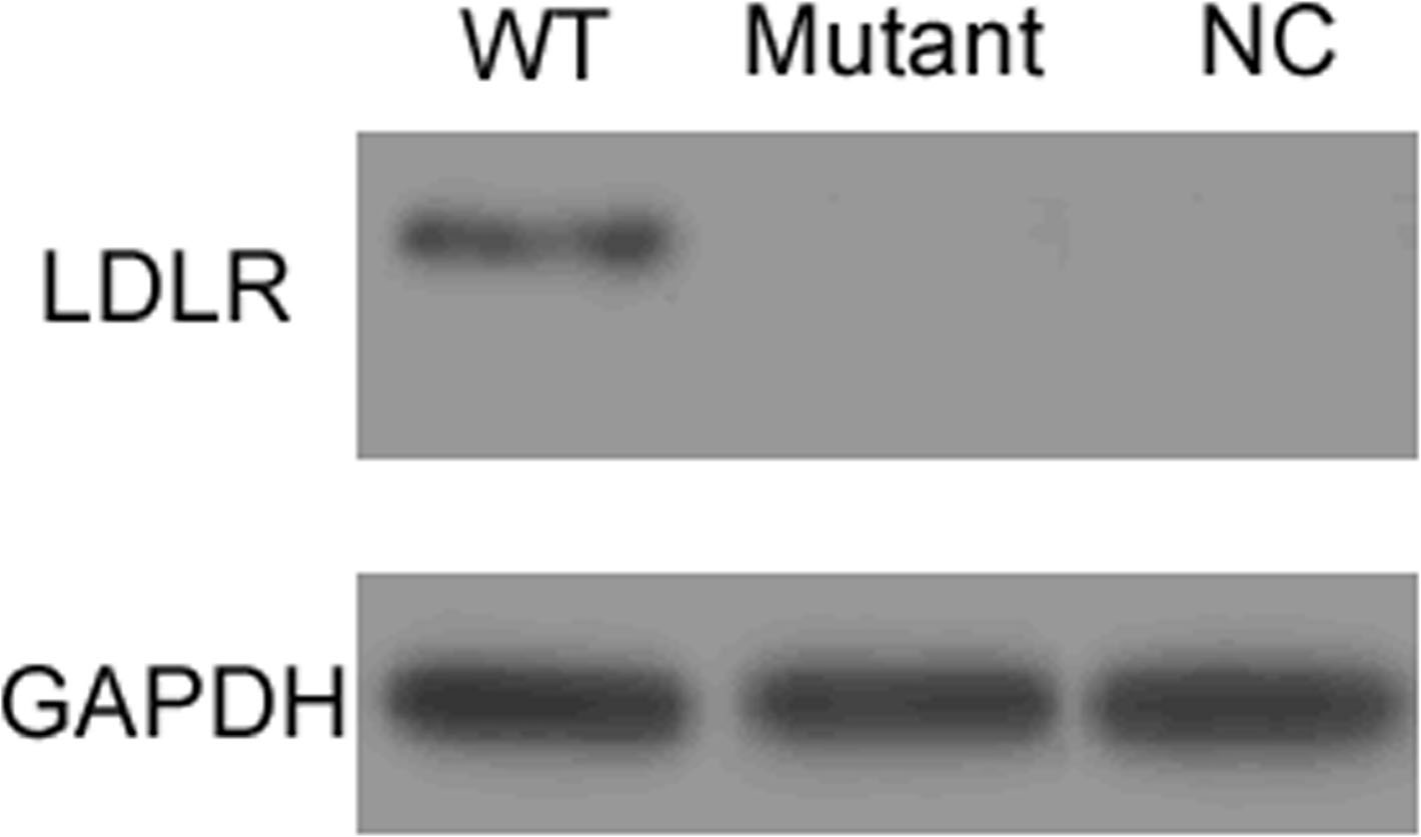
The protein expression of LDLR c.501C>A in HEK293 cells

The levels of LDLR protein in HEK293 cells transfected with the wide-type, mutant variant (*LDLR* c.501C>A) and empty plasmids were determined by Western Blotting analysis. The LDLR is normally expressed in WT cells (Fig. 2). The expression of LDLR was null in cells transfected with the plasmid containing the mutant variant or cells transfected with the empty plasmid. These results suggested that the disease-causing variant *LDLR* c.501C>A prevented the LDLR protein from being expressed normally in the cells.

### *LDLR* c.501C>A variant prevents Dil-LDL uptake in HEK293 cells

**Figure 3:**
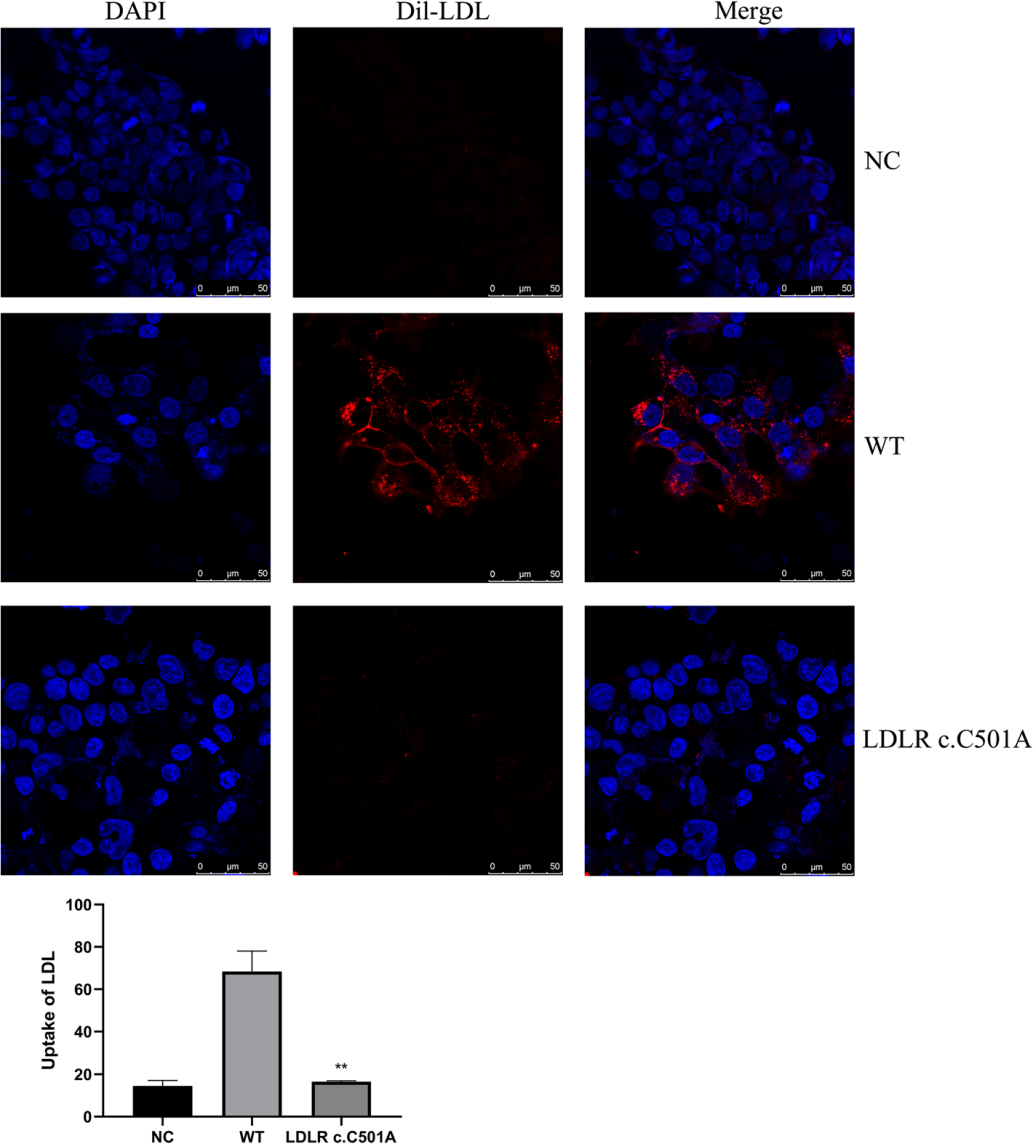
The representative confocal microscopy images of cell-associated DAPI (blue), Dil-LDL (red) and the merge. The data represent means ± SEM. **P < 0.01 vs. WT

The ability to uptake Dil-LDL was assayed in HEK293 cells expressing wide-type *LDLR* or *LDLR* c.501C>A. The uptake of LDL in cells with *LDLR* c.501C>A was highly decreased compared with the cells carrying the wide-type *LDLR* gene (Fig. 3, *P* < 0.01). No statistically significant difference was observed in the uptake of Dil-LDL between cells transfected with empty plasmid and mutant plasmid.

### *LDLR* c.501C>A is a disease-causing variant for FH

Loss of function (LOF) of LDLR is one of the known pathogenic mechanisms of FH. In this study, we used the ACMG standards and guidelines to determine the pathogenicity of *LDLR* c.501C>A variant. According to the ACMG criteria, *LDLR* c.501C>A is a disease-causing variant for familial hypercholesterolemia with very strong evidence of pathogenicity (PVS1).

### PCSK9 inhibitor therapy is not highly effective for FH patients with *LDLR* c.501C>A variant

Before this study, proband 1 and proband 2 still did not reach the lipid lowering treatment goal after receiving high doses of statins and ezetimibe. Therefore, PCSK9 inhibitor (Evolocumab Injection, 140mg Q2W) therapy was initiated on the two probands. The patients were followed up for 3 months to evaluate their response to the treatment.

**Figure 4:**
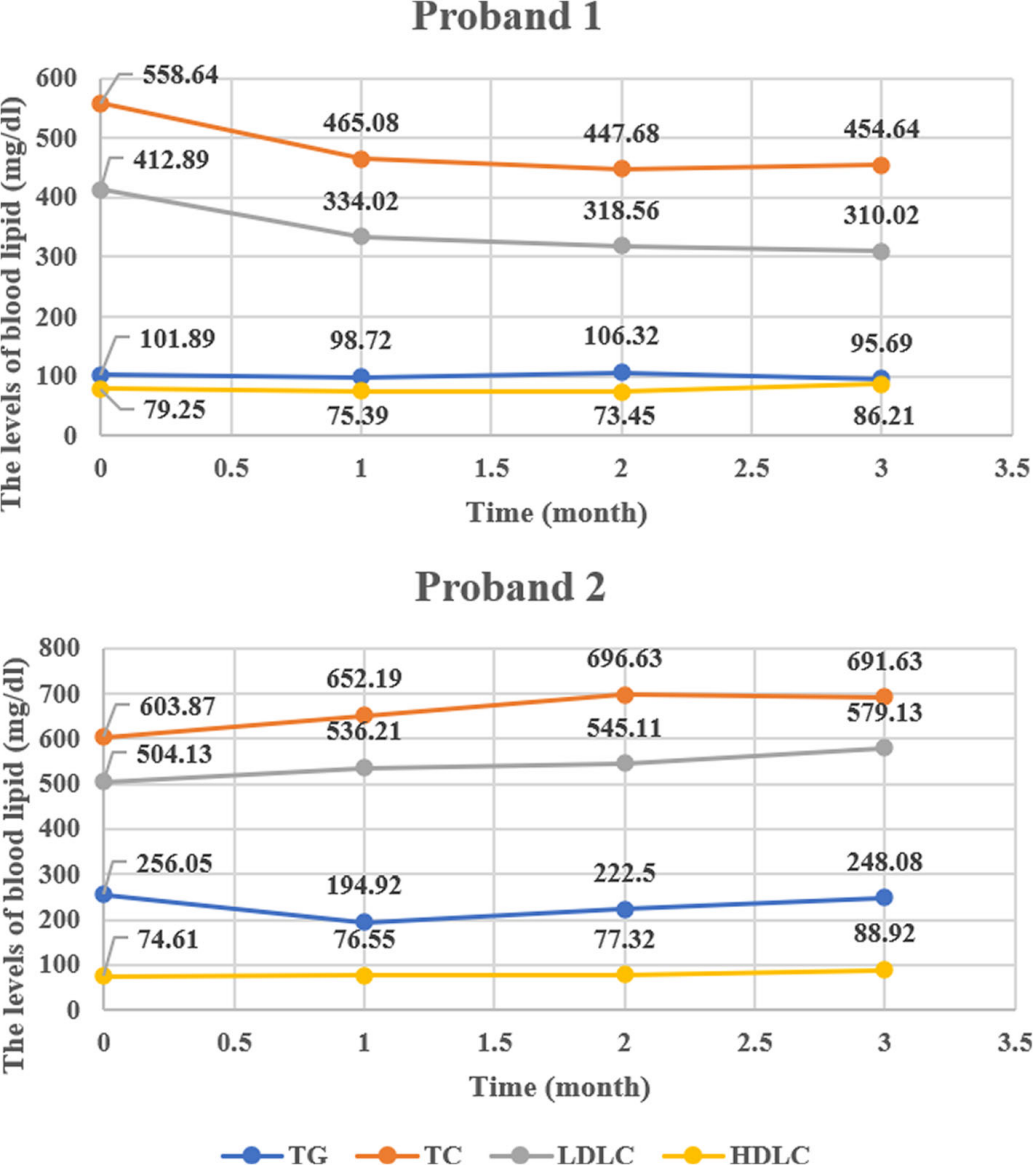
The blood lipid levels of two probands before and after PCSK9 inhibitor treatment

As shown in Fig. 4, the LDLC level of proband 1 was significantly reduced from 412.89 to 310.02 mg/dl (24.91%). However, the reduction was far from the expected effect. In contrast to proband 1, PCSK9 inhibitor treatment had no lipid-lowering effect for the proband 2. Rather, the LDLC level was slightly increased. The follow-up results showed that the PCSK9 inhibitor treatment was not highly effective for two probands.

## Discussion

In general, LDLR-mediated LDL endocytosis plays a key role in regulating plasma cholesterol concentration and cholesterol homeostasis. In the current study, the disease-causing variant *LDLR* c.501C>A was observed in two FH families. Its function was verified by bioinformatics analysis and *in vitro* cell experiments. In addition, the effect of lipid-lowering treatment in FH patients was assessed for 3 months.

*LDLR* gene (Ch19 pl3.1-13.3) consists of 18 exons and 17 introns [23]. Based on the synthesis and function of LDLR protein, mutations could be divided into 5 types [24]. Type 1 mutation is a non-expression allele, including promoter sequence mutation, nonsense mutation, frame-shift mutation, and splicing mutation, which lead to non-expression of *LDLR* gene. In this study, we found that the *LDLR* c.501C>A variant significantly affected the blood lipid levels in patients. Whole-exome sequencing revealed that *LDLR* c.501C>A is a nonsense mutation within exon 4, which belongs to type 1 mutation. This type of mutation prevented the *LDLR* protein from being expressed normally as confirmed with cell experiments. The abnormal expression of LDLR due to *LDLR* c.501C>A explains the reduced uptake of LDL and eventually abnormal lipid metabolism.

The severity of hypercholesterolemia in FH is not only associated with the function of the mutant protein but also on the number of defective alleles [25]. On the basis of the patients’ blood lipid level and clinical symptoms, both probands were suspected as HoFH. According to the molecular genotype, HoFH patients can be divided into true homozygotes, compound heterozygotes, and double heterozygotes. Interestingly, the sequencing results showed that the 1-I-2, 1-II-1, and 2-III-1 patients were heterozygous, but 2-II-3 was HoFH. The results suggested that the genotype and phenotype of FH patients (especially proband 1) were not consistent. Furthermore, the cholesterol levels of proband 1’s son (1-III-1) were high without any suspicious variant being detected. This could be due to the fact that this study focused on the variants in the classic FH-related genes. It could not rule out the possibility that there are other undiscovered variants in other related genes. Besides, there may be other pathologies related to the genetic condition of this family. Further studies are required to confirm these hypotheses.

Distinguishing homozygotes from heterozygotes based on clinical manifestations alone is not always practically possible. Genetic testing is the "gold standard" for diagnosing FH. The accurate diagnosis requires an accurate assessment of the clinical manifestations and DNA sequences of patients and their family members. Unfortunately, the diagnosis rate in most countries is < 1%, and the treatment status is even worse [26]. Therefore, it is of great significance to carry out reasonable population screening, improve the diagnosis rate of FH, and implement standardized treatment and long-term management [27].

The response of FH patients to drugs varies greatly. The type of LDLR variant is an important predictor of the response to lipid-lowering therapy in the FH population [28]. Mutant alleles can generally be divided into two types, null alleles and defective alleles [29]. FH patients with null alleles had worse response to lipid lowering treatment, and the risk of ASCVD was also significantly increased in the patients [30]. The TESLA Part B study showed that when treated with the PCSK9 inhibitor, patients with at least one defective LDLR allele had an LDLC reduction of 41%. However, patients with two defective alleles had an LDLC reduction of 47%. The LDLC in patients with LDL receptor deficiency/null status was reduced by 25%. In one patient with LDLR null/null status, LDLC increased by 10% under Evolocumab treatment [31]. All the current research findings support the use of genetics to manage FH more effectively in the future.

With regard to the *LDLR* c.501C>A variant, proband 1 was heterozygous while proband 2 was homozygous. When PCSK9 inhibitor was applied to both probands, their responses were not consistent. After 3 months of follow-up, it was found that the LDLC level of proband 1 reduced by 24.91% from the original level. Similar to previous reports inhibitor [31], proband 2 with HoFH showed no response to the PCSK9 inhibitor. Thedrez *et al.* found that the residual expression of LDLR in HoFH determined the patients’ LDLC level and their response to PCSK9 inhibitor [32]. However, the relationship between genotype, treatment effect, and response mechanism should be thoroughly studied.

### Study strength and limitations

In this study, it was confirmed that *LDLR* c.501C>A, a nonsense mutation, caused loss of gene function by affecting protein expression. In addition, the poor response to treatment with PCSK9 inhibitors was observed in two probands. These results suggest that personalized treatment plans should be developed to improve the response of FH patients to treatment. Therefore, the current research provides a basis for use of genotype to guide the treatment of FH patients in the future. The current research had some limitations. First, only two FH families participated in this study. More FH patients need to be studied to understand the full spectrum of disease-causing variants among FH patients in China. Secondly, the study confirmed the effect of the variants at the cellular level only. These results should be confirmed with *in vivo* studies in the future. Finally, the study mainly focused on variants within the exon regions of the *LDLR* gene. Future studies should be performed to explore variants within the intronic region of *LDLR, APOB, PCSK9* genes or large structural variants in *LDLR* gene and establish their effect on the occurrence and development of FH disease.

## Conclusion

In conclusion, *LDLR* c.501C>A might be a disease-causing variant for FH. Moreover, PCSK9 inhibitors were not ideal for lowering lipids in FH patients with *LDLR* c.501C>A variant. In the future, it is still necessary to develop new treatments to improve the symptoms and survival of FH patients. A good example could be the gene editing technology to correct this variant.

## Supplementary Information

**Additional file 1: Supplement Figure** 1. Target sequences on LDLR by Sanger sequencing. The top row of the sequence represented the original sequence, and the second row showed the mutant sequence. And the framed base indicated the mutation (*LDLR* c.501C>A).

**Additional file 2: Supplementary Table** 1. The variants of the nucleotide sequence in *LDLR, APOB, PCSK9* genes with MAF less than 0.05 in the family of Proband 1.

## Data Availability

The data that support the findings of this study are available from the corresponding author upon reasonable request.

## Abbreviations

FH: Familial hypercholesterolemia
LDLC: low-density lipoprotein cholesterol
ASCVD: atherosclerotic cardiovascular disease
HeFH: Heterozygous FH
HoFH: homozygous FH
LDLR: low-density lipoprotein receptor
ApoB: apolipoprotein B
PCSK9: proprotein convertase subtilisin kexin 9
LDLRAP1: low-density lipoprotein receptor adapter protein 1
DLCN: Dutch Lipid Clinic Network
FBS: fetal bovine serum
WT: wild type
ACMG: American College of Medical Genetics and Genomics
HDLC: high-density lipoprotein cholesterol
