# Supplementary figures and images for: The LDLR c.501C>A is a disease-causing variant in familial hypercholesterolemia
Source: Lipids Health Dis. 2021 Sep 12;20:101. doi: 10.1186/s12944-021-01536-3 (PMC8436568; doi:10.1186/s12944-021-01536-3)

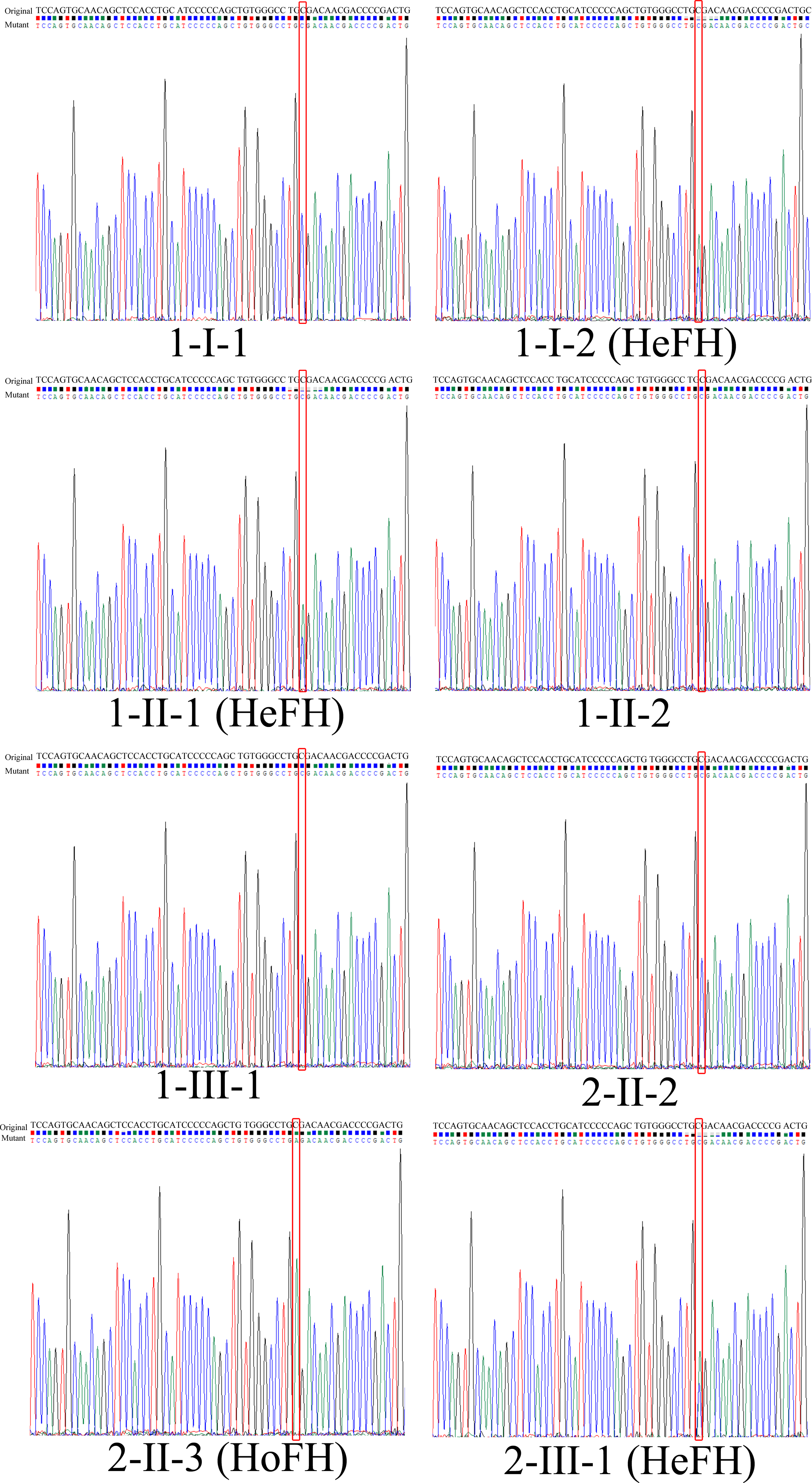

Supplement: Supplementary file 1 — Additional file 1: Supplement Figure 1. Target sequences on LDLR by Sanger sequencing. The top row of the sequence represented the original sequence, and the second row showed the mutant sequence. And the framed base indicated the mutation (LDLR c.501C>A). [file 12944_2021_1536_MOESM1_ESM.tif]
