# Supplementary material for: The LDLR c.501C>A is a disease-causing variant in familial hypercholesterolemia
Source: Lipids Health Dis. 2021 Sep 12;20:101. doi: 10.1186/s12944-021-01536-3 (PMC8436568; doi:10.1186/s12944-021-01536-3)
Supplement: Supplementary file 2 — Additional file 2: Supplementary Table 1. The variants of the nucleotide sequence in LDLR, APOB, PCSK9 genes with MAF less than 0.05 in the family of Proband 1. [file 12944_2021_1536_MOESM2_ESM.docx]

Supplementary Table 1: The variants of the nucleotide sequence in LDLR, APOB, PCSK9 genes with MAF less than 0.05 in the family of Proband 1.

| Gene | Filter | Function | Chr. | Start | End | Ref | Alt | 1-I-1 | 1-I-2 | 1-II-1 | 1-II-2 | 1-III-1 |
| --- | --- | --- | --- | --- | --- | --- | --- | --- | --- | --- | --- | --- |
| PCSK9 | not_exonic | intronic | chr1 | 55505804 | 55505804 | T | A | 0/0 | 0/1 | 0/1 | 0/0 | 0/1 |
| PCSK9 | not_exonic | intronic | chr1 | 55510015 | 55510015 | C | T | 0/0 | 0/1 | 0/1 | 0/0 | 0/0 |
| PCSK9 | not_exonic | intronic | chr1 | 55526840 | 55526840 | C | G | 0/1 | 0/0 | 1/1 | 0/0 | 0/0 |
| APOB | not_exonic | intronic | chr2 | 21266164 | 21266164 | C | T | 0/1 | 0/0 | 0/1 | 0/0 | 0/0 |
| LDLR | not_exonic | intronic | chr19 | 11211079 | 11211079 | C | T | 0/1 | 0/0 | 0/1 | 0/0 | 0/1 |
| LDLR | not_exonic | intronic | chr19 | 11213689 | 11213689 | G | A | 0/1 | 0/0 | 0/1 | 0/0 | 0/1 |
| LDLR | deleterious | exonic | chr19 | 11216083 | 11216083 | C | A | 0/0 | 0/1 | 0/1 | 0/0 | 0/0 |
